# Subjective symptoms are associated with anxiety in patients with rosacea: a cross-sectional study

**DOI:** 10.3389/fpubh.2026.1886167

**Published:** 2026-08-13

**Authors:** Yuwei Huang, Xu Liu, Yingying Dai, Lian Wang, Lu Zhang, Xian Jiang

**Affiliations:** 1 Department of Dermatology, West China Hospital, Sichuan University, Chengdu, China; 2 Laboratory of Dermatology, Clinical Institute of Inflammation and Immunology, Frontiers Science Center for Disease-related Molecular Network, West China Hospital, Sichuan University, Chengdu, China

**Keywords:** anxiety, carvedilol, life quality, rosacea, treatment

## Abstract

**Background:**

Rosacea, a chronic inflammatory skin disorder, affects patients’ physical health. The risk of anxiety disorders in patients with rosacea may be 2–3 times higher than that in the general population. However, the key factors influencing anxiety in rosacea remain unclear, and whether alleviating the subjective symptoms of rosacea can further improve treatment outcomes and quality of life requires further investigation.

**Methods:**

A retrospective cross-sectional study of 482 newly diagnosed rosacea patients (West China Hospital) was conducted, with grouping into anxiety/non-anxiety groups for risk factor analysis. After that, a 6-week sub-analysis compared 64 patients receiving conventional carvedilol therapy vs. 32 receiving carvedilol + subjective symptom-targeted treatment, using univariate/multivariate logistic regression and pre−/post-treatment symptom/quality-of-life/psychological status comparisons.

**Results:**

Patients with erythematotelangiectatic rosacea (ETR) had significantly higher Hospital Anxiety and Depression Scale-Anxiety (HADS-A) scores (*p* = 0.03). Multivariate analysis revealed that moderate-to-severe burning sensation (odds ratio [OR] = 1.63), dryness (OR = 1.90), and pruritus (OR = 2.76) were independent risk factors for anxiety (all *p* < 0.05). Compared with the conventional group, the targeted group exhibited superior improvements in Investigator’s Global Assessment (IGA) scores (*p* = 0.025), Patient Self-Assessment (PSA) scores (*p* = 0.002), subjective symptoms, HADS-A scores (*p* = 0.002), and Dermatology Life Quality Index (DLQI) scores (*p* = 0.033). patients with erythematotelangiectatic rosacea (ETR) had significantly higher.

**Conclusion:**

Subjective symptoms are independently associated with rosacea-related anxiety. Targeted treatment addressing subjective symptoms is associated with improvements in both overall clinical outcomes and psychological status, highlighting the necessity of systematic assessment and management of subjective symptoms in clinical practice.

## 1 Introduction

Rosacea is a chronic inflammatory skin disorder that primarily affects the mid-face, with a global prevalence of approximately 5.46% (1). It is characterized by flushing, erythema, papules, pustules, and telangiectasia (2). Notably, this disorder often entails psychiatric comorbidities like anxiety and depression, exacerbating disease burden (3). A meta-analysis involving 14,134,021 rosacea patients revealed that the pooled prevalence of depression among rosacea patients was 19.6%, and that of anxiety was 15.6% (4). Moreover, compared with the general healthy population, the incidence of anxiety disorders was significantly higher in rosacea patients (5, 6). Paying attention to the psychological characteristics of rosacea patients can improve their overall well-being (7, 8). Growing attention has been paid to the correlation between rosacea and neural regulation; however, many patients fail to respond to psychiatric treatments (9). This highlights the necessity of refining patient subtyping and attaching greater importance to overlooked minor or subjective symptoms.

Beyond visually apparent symptoms, patients with rosacea may also experience subjective symptoms such as burning sensation, stinging, dryness, and pruritus (9). Subjective symptoms are highly common in rosacea patients; burning and stinging sensations may occur across all subtypes (10). In 2013, Scharschmidt et al. (11) proposed the concept of neurogenic rosacea (NR). Compared with the observed degree of erythema, the subjective symptoms of NR patients are usually disproportionately severe, and such NR patients are often accompanied by other neurological and psychiatric comorbidities. Existing studies have shown a significant correlation between the reduction in burning/stinging sensations and improvements in an individual’s quality of life (10). These previous studies all suggest that subjective symptoms in rosacea patients may be associated with their psychological status and quality of life (12). Clinically targeted symptomatic treatment to alleviate patients’ symptoms may not only improve their physical symptoms but also enhance their quality of life and ameliorate their psychological state (13).

Therefore, this retrospective study aims to analyze the psychological and emotional characteristics of rosacea patients, and to explore whether treatments targeting subjective symptoms can alleviate patients’ symptoms while improving their overall physical and psychological status. The significance of this study lies in providing a basis for the targeted relief of subjective symptoms, thereby enhancing patients’ quality of life and treatment efficacy, and filling the gap in clinical attention to the interaction between psychological status and subjective symptoms.

## 2 Methods

### 2.1 Study design and patient enrollment

A retrospective cross-sectional study design was adopted. Patients with rosacea who attended the Outpatient Department of Dermatology at West China Hospital of Sichuan University from May 31, 2024, to June 1, 2025, were enrolled retrospectively.

Inclusion criteria were as follows:

i) All enrolled patients were diagnosed with rosacea by dermatologists in accordance with the 2019 Rosacea Consensus (ROSCO) (14, 15)
ii) Newly diagnosed patients or those who had not received treatment in the past 3 months;
iii) Adults (≥18 years old);
iv) Sufficient literacy to describe their own symptoms in detail.
v) Exclusion criteria were as follows:
vi) Patients with incomplete data or inability to cooperate with follow-up;
vii) Patients concurrently suffering from other skin diseases or systemic diseases that might interfere with the phenotypic diagnosis of rosacea;
viii) Minors, pregnant women, or patients in other special physiological states;
ix) Patients undergoing treatment for other diseases (e.g., oral medication, topical medication, surgical treatment, etc.).

This study was approved by the Ethics Committee of West China Hospital and conducted in compliance with the Declaration of Helsinki.

### 2.2 Establishment and matching of the symptomatic treatment group

Given the non-randomized nature of this observational sub-analysis, we emphasize that all findings from this comparison should be interpreted as associations rather than causal therapeutic effects. To minimize confounding, we performed rigorous 1:2 matching on key baseline characteristics.

To explore whether the alleviation of subjective symptoms is beneficial for the overall disease condition and anxiety status of rosacea patients, screening was conducted among the enrolled patients. Oral carvedilol (5 mg, three times a day [t.i.d]) was used as the main conventional treatment regimen (16). Patients were screened if they received symptomatic treatment for subjective symptoms (burning sensation, stinging, dryness, pruritus) (e.g., methylcobalamin or antihistamines) in addition to the conventional carvedilol treatment.

Inclusion criteria for the symptomatic treatment group were:

- i) Receiving oral carvedilol (5 mg, t.i.d) as the treatment regimen while concurrently undergoing symptomatic treatment for subjective symptoms (burning sensation, stinging, dryness, pruritus);
- ii) Receiving rosacea medication treatment for the first time, or having discontinued other medications for more than 3 months;
- iii) Adhering to this treatment regimen and completing regular follow-up visits and re-examinations at the hospital for 6 weeks or longer.

1:2 intra-cohort matching was conducted on patients included in the symptomatic treatment group to form a control group. The matching criteria for the control group were as follows:

- i) Matching gender and age (±5 years) with patients in the symptomatic treatment group;
- ii) Matching subjective and objective symptoms of rosacea with patients in the symptomatic treatment group;
- iii) Receiving oral medication for the first time or having discontinued other medications for more than 3 weeks;
- iv) Receiving oral carvedilol (5 mg, t.i.d.) as the treatment regimen, and maintaining the regimen for more than 6 weeks.

### 2.3 Evaluation indicators

The classification of rosacea subtypes referred to the definition proposed by Crawford et al., which classifies rosacea into 4 broad subtypes: erythematotelangiectatic rosacea (ETR), papulopustular rosacea (PPR), phymatous rosacea (PhR), and ocular rosacea (OR). This study focused on cutaneous rosacea and did not include patients with ocular rosacea. For the phenotypic assessment of rosacea, the 2019 Rosacea Consensus (ROSCO) was followed. Phenotypic symptoms were graded into 5 levels: Grade 0 (no symptoms), Grade 1 (almost no symptoms), Grade 2 (mild), Grade 3 (moderate), and Grade 4 (severe). A patient was considered to have moderate-to-severe symptoms of a specific phenotype if their score for that phenotype was 3 or 4. In addition, based on the overall condition of patients, the Investigator’s Global Assessment (IGA) score, Patient’s Self-Assessment (PSA) score, and Clinician’s Erythema Assessment (CEA) score were used to evaluate rosacea. All scores ranged from 0 to 4, with higher scores indicating more severe overall rosacea.

For the assessment of patients’ psychological status, the Hospital Anxiety and Depression Scale (HADS) was employed to evaluate the levels of anxiety and depression. In both the Depression Subscale (HADS-D) and the Anxiety Subscale (HADS-A), A score of 0–7 was considered negative; 8–10 indicated mild anxiety/depression; 11–14 indicated moderate anxiety/depression; 15–21 indicated severe anxiety/depression (17). The Dermatology Life Quality Index (DLQI) was used to assess patients’ quality of life, with the following scoring criteria: 0–1 point: No impact of the disease on life; 2–5 points: Mild impact; 6–10 points: Moderate impact; 11–20 points: Severe impact; 21–30 points: Extremely severe impact (18).

### 2.4 Statistical methods

Quantitative variables are expressed as mean ± standard deviation (SD), while categorical data are presented as frequencies and percentages. Categorical variables are assessed using Fisher’s exact test or the χ^2^ test. All ordinal variables are analyzed using the Mann–Whitney *U* test or Kruskal-Wallis *H* test. For continuous variables: Those conforming to a normal distribution are analyzed using the independent samples *t*-test; Those not conforming to a normal distribution are analyzed using the Mann–Whitney *U* test. Univariate analysis and multiple logistic regression analysis were conducted to calculate odds ratios (OR) and their 95% confidence intervals (CI).

For the multivariable logistic regression, variables with a *p*-value <0.1 in the univariate analysis were considered for entry into the final model. To assess potential multicollinearity among the independent variables, particularly the subjective symptom domains (burning, stinging, dryness, pruritus), we calculated the Variance Inflation Factor (VIF). A VIF threshold of <5 was used to indicate no significant collinearity.

For subgroup comparisons involving small sample sizes, results were interpreted with caution and primarily considered as descriptive statistics, given the limited statistical power. All reported *p*-values are based on two-sided tests, with a significance level of *α* = 0.05. All statistical analyses were performed using SPSS version 28.0.

## 3 Results

### 3.1 Patient enrollment results and demographic characteristics

Based on the comprehensive application of inclusion and exclusion criteria, a total of 482 patients with cutaneous rosacea were enrolled in this study. Among them, 410 patients were primarily classified as erythematotelangiectatic rosacea (ETR), 62 as papulopustular rosacea (PPR), and 10 as phymatous rosacea (PhR). The baseline demographic characteristics of the patients are presented in Table 1. No statistically significant differences were observed in the demographic characteristics among the three subtypes. However, patients with ETR rosacea exhibited a higher level of anxiety as evaluated by the HADS-A scale (*p* = 0.03).

**Table 1:** Demographic baseline of rosacea patients included in the study.

Characteristic	ETR (410)	PPR (62)	PhR (10)	*p*-value
Sex				
Male	154 (37.6%)	23 (37.1%)	4 (40.0%)	0.92
Female	256 (62.4%)	39 (62.9%)	6 (60.0%)	
Age	40.58 ± 11.20	40.72 ± 11.05	41.2 ± 11.5	0.95
BMI	22.7 ± 4.8	22.8 ± 4.7	22.6 ± 5.0	0.98
DLQI				
None	17 (4.1%)	2 (3.2%)	1 (10.0%)	0.87
Mild	51 (12.4%)	8 (12.9%)	1 (10.0%)	
Moderate	89 (21.7%)	13 (21.0%)	2 (20.0%)	
Severe	164 (40.0%)	24 (38.7%)	5 (50.0%)	
Extremely severe	89 (21.7%)	15 (24.2%)	1 (10.0%)	
Anxiety (HADS-A)				
None	97 (23.7%)	23 (37.1%)	3 (30.0%)	**0.03**
Mild	53 (13.0%)	11 (17.7%)	2 (20.0%)	
Moderate	136 (33.2%)	14 (22.6%)	2 (20.0%)	
Severe	124 (30.2%)	14 (22.6%)	3 (30.0%)	
Depression (HADS-D)				
None	124 (30.2%)	19 (30.6%)	4 (40.0%)	0.81
Mild	94 (22.9%)	14 (22.6%)	2 (20.0%)	
Moderate	102 (24.9%)	15 (24.2%)	3 (30.0%)	
Severe	90 (21.9%)	14 (22.6%)	1 (10.0%)	

Bold indicates *p*-values with statistical significance (*p* < 0.05).

### 3.2 Factors associated with anxiety in patients with Rosacea

To investigate the differences in anxiety levels among rosacea patients, the 482 patients were divided into two groups based on the HADS-A scale ratings: the non-anxious group (HADS-A scale rating indicating no anxiety, *n* = 123) and the anxious group (HADS-A scale rating indicating mild, moderate, or severe anxiety, *n* = 359).

Univariate analysis revealed no significant differences in demographic characteristics such as gender, age, and BMI between the two groups. However, significant differences were observed in the following clinical phenotypes of rosacea: telangiectasia (*p* = 0.005), persistent erythema (*p* = 0.077), burning sensation (*p* = 0.007), stinging (*p* = 0.005), edema (*p* = 0.009), dryness (*p* = 0.001), and pruritus (*p* = 0.007). Additionally, according to the DLQI ratings, the anxious group demonstrated a lower quality of life compared to the non-anxious group (*p* < 0.001) (Supplementary Table 1).

Factors with statistical significance identified in the univariate analysis were included in the multivariate analysis. The results showed that for rosacea patients, visible symptoms such as moderate-to-severe telangiectasia (OR, 95% CI: 1.31, 0.74–2.33; *p* = 0.342) and moderate-to-severe persistent erythema (OR, 95% CI: 1.17, 0.67–2.04; *p* = 0.578) were not associated with anxiety. In contrast, subjective symptoms—specifically moderate-to-severe burning sensation (OR, 95% CI: 1.63, 1.07–2.48; *p* = 0.016), moderate-to-severe dryness (OR, 95% CI: 1.90, 1.01–3.57; *p* = 0.045), and moderate-to-severe pruritus (OR, 95% CI: 2.76, 1.42–5.37; *p* = 0.003), were independent risk factors for the development of anxiety in these patients (Table 2).

**Table 2:** Risk factors of anxiety in rosacea patients according to the multivariate analysis^†^.

Characteristic	Without anxiety (123)	With anxiety (359)	OR (95% CI)	*p*-value
Telangiectasia				
None or mild	104 (84.6%)	259 (72.0%)		0.342
Moderate to severe	19 (15.4%)	100 (28.0%)	1.31 (0.74–2.33)	
Persistent erythema				
None or mild	24 (19.7%)	47 (13.1%)		0.578
Moderate to severe	98 (80.3%)	312 (86.9%)	1.17 (0.67–2.04)	
Burning				
None or mild	88 (71.5%)	208 (57.9%)		**0.016**
Moderate to severe	35 (28.5%)	151 (42.1%)	1.63 (1.07–2.48)	
Stinging				
None or mild	118 (95.9%)	312 (86.9%)		0.223
Moderate to severe	5 (4.1%)	47 (13.1%)	1.87 (0.68–5.15)	
Edema				
None or mild	118 (95.9%)	315 (87.7%)		0.281
Moderate to severe	5 (4.1%)	44 (12.3%)	1.72 (0.64–4.65)	
Dryness				
None or mild	113 (91.9%)	297 (77.9%)		**0.045**
Moderate to severe	10 (8.1%)	79 (22.1%)	1.90 (1.01–3.57)	
Pruritus				
None or mild	116 (94.3%)	305 (85.0%)		**0.003**
Moderate to severe	7 (5.7%)	54 (15.0%)	2.76 (1.42–5.37)	

^†^Reference group for each logistic regression model are without anxiety (*n* = 123); the cases with unknown characteristics are excluded.

Bold indicates *p*-values with statistical significance (*p* < 0.05).

### 3.3 Symptomatic treatment for subjective symptoms in Rosacea patients

Treatment screening was performed on patients included in the cohort, and 32 patients were enrolled in the symptomatic treatment group. A 1:2 matching was performed for the 32 patients in the symptomatic treatment group, and 64 patients receiving carvedilol treatment were included as the conventional treatment control group.

The baseline characteristics of the two groups are shown in Table 3. No significant statistical differences were observed between the two groups in terms of baseline demographics, treatment duration, severity of rosacea symptoms, psychological status or quality of life (all *p* > 0.05), indicating good homogeneity between the two groups (Table 3).

**Table 3:** Baseline characteristics of rosacea patients treated by carvedilol only and carvedilol plus subjective symptom-related drugs.

Characteristic	Carvedilol group (*n* = 64)	CVD+ subjective symptoms-related drugs group (*n* = 32)	*p*-value
Sex			
Male	13 (20.3%)	8 (25.0%)	0.603
Female	51 (79.7%)	24 (75.0%)	
Age	42.44 (1.61)	43.13 (1.93)	0.058
BMI	23.05 (3.61)	24.04 (3.70)	0.297
Course of disease (months)	13.77 (4.42)	14.32 (3.93)	0.537
Course of treatment (weeks)	8.85 (2.12)	9.04 (2.58)	0.713
IGA	2.75 (0.73)	2.71 (0.73)	0.844
CEA	2.68 (0.69)	2.65 (0.70)	0.835
PSA	2.60 (0.78)	2.56 (0.80)	0.985
Burning	2.65 (0.78)	2.65 (0.78)	1
Stinging	1.20 (0.91)	1.31 (1.06)	0.61
Dryness	1.09 (1.30)	1.29 (1.35)	0.485
Pruritus	1.57 (1.23)	1.78 (1.16)	0.439
HADS-A	8.76 (5.14)	8.75 (5.00)	0.989
HADS-D	5.12 (3.11)	4.43 (3.79)	0.346
DLQI	8.64 (5.25)	10.71 (5.63)	0.078

A comparison of the improvement in various indicators before and after treatment between the conventional treatment group (carvedilol alone) and the group receiving additional symptomatic treatment for subjective symptoms showed that after more than 6 weeks of treatment, the improvement in IGA scores and PSA scores in the group with additional symptomatic treatment was significantly higher than that in the conventional treatment group (*p* = 0.025, *p* = 0.002). This indicates that adding treatment targeting subjective symptoms to the conventional treatment regimen can improve the overall symptoms of patients. Meanwhile, symptoms such as burning sensation (*p* < 0.001), dryness (*p* = 0.018), and pruritus (*p* < 0.001) were more alleviated in the group with additional symptomatic treatment compared to the conventional treatment group. In addition, the improvements in HADS-A scores, HADS-D scores, and DLQI scores in the group with additional symptomatic treatment were superior to those in the conventional treatment group (*p* = 0.002, *p* = 0.023, *p* = 0.033), suggesting that symptomatic treatment for subjective symptoms leads to better improvements in patients’ psychological status and quality of life (Table 4).

**Table 4:** Comparison of therapeutic efficacy of rosacea between carvedilol group and carvedilol plus subjective symptom-related drugs group.

Characteristic	Carvedilol group (*n* = 64)	CVD+ subjective symptoms-related drugs group (*n* = 32)	*p*-value
IGA	0.49 (0.99)	0.90 (0.90)	**0.025**
CEA	0.76 (0.96)	0.78 (0.92)	0.835
PSA	0.34 (1.15)	0.44 (0.97)	**0.002**
Burning	0.58 (0.99)	1.56 (1.25)	**<0.001**
Stinging	0.55 (1.27)	0.75 (1.35)	0.75
Dryness	0.50 (1.62)	0.70 (1.44)	**0.018**
Pruritus	0.67 (1.51)	1.10 (1.40)	**<0.001**
HADS-A	2.01 (4.14)	4.25 (3.83)	**0.002**
HADS-D	1.78 (2.92)	2.18 (3.19)	**0.023**
DLQI	1.71 (4.81)	4.37 (5.10)	**0.033**

Bold indicates *p*-values with statistical significance (*p* < 0.05).

Graphical abstracts should contain a clear and original image that accurately represents either the results or the working hypothesis described in the article.

The authors may create relevant graphical representations for the graphical abstract and they may also reuse the figures from the article.

## 4 Discussion

This study conducted a retrospective cross-sectional analysis of 482 patients with cutaneous rosacea to explore the association between subjective discomfort symptoms and anxiety, and the efficacy of targeted symptomatic treatment. We found that subjective discomfort symptoms (moderate-to-severe burning sensation, dryness, and pruritus) are independent risk factors for anxiety in rosacea patients, while targeted treatment for these symptoms significantly improves overall disease status, anxiety levels, and quality of life, provide critical insights for refining clinical management of rosacea and addressing the psychological comorbidities of this chronic skin disorder.

Consistent with previous meta-analyses reporting a 15.6% anxiety prevalence in rosacea patients, our study further identified the specific clinical factors underlying this psychological burden. Notably, multivariate analysis revealed that visible phenotypic symptoms were not associated with anxiety, whereas subjective symptoms (burning sensation, dryness, and pruritus) emerged as independent risk factors (OR = 1.63, 1.90, 2.76, respectively). Importantly, patients with such subjective symptoms were often accompanied by not only psychiatric burden but also poorer quality of life. It is worth noting that this finding is closely related to the association between ETR and anxiety. Specifically, different rosacea subtypes have distinct pathological focuses, PPR primarily features inflammatory responses, PhR is characterized by local hypertrophy and hyperplasia, while ETR and anxiety share common mechanisms in neurovascular regulatory disorders. Moreover, anxiety and persistent erythema may interact reciprocally, a cycle that impairs treatment efficacy. This highlights the clinical significance of prioritizing the management of subjective symptoms and psychological status in ETR patients. The choice of methylcobalamin in our symptomatic treatment group was based on its recognized role as a neurotrophic agent, supporting peripheral nerve repair and function. In the context of rosacea, it is empirically used to alleviate neuropathic sensory symptoms like burning and stinging, which are hallmarks of neurogenic rosacea. However, we acknowledge that robust clinical trial evidence for methylcobalamin in rosacea is currently limited. Other neuromodulatory agents, such as gabapentin and pregabalin, have also been reported in the literature for the management of neurogenic rosacea and refractory facial sensory symptoms, offering alternative options for patients with severe neuropathic features. Notably, the observed association between the combined regimen and better outcomes should be interpreted with caution, as the non-randomized allocation may have been influenced by unmeasured confounders, such as the severity of sensory symptoms at baseline or physician prescribing preferences.

The mechanistic link between subjective symptoms and anxiety may stem from the persistent, sensory nature of these symptoms (19). From a pathophysiological standpoint, this link is supported by the concept of neurogenic inflammation in rosacea. Activation of sensory nerve endings, particularly through transient receptor potential (TRP) channels like TRPV1 and TRPA1, can be triggered by various rosacea stimuli. This activation leads to the release of neuropeptides such as substance P and calcitonin gene-related peptide (CGRP), which not only mediate vasodilation but also promote inflammation and sensitize nociceptors, amplifying sensations of burning and stinging. This neuro-vascular-inflammatory axis, when chronically activated, could create a persistent state of sensory hypervigilance. The unrelenting and unpredictable nature of these intrusive sensations may, over time, foster a cycle of anxiety and worry, particularly when patients feel their invisible symptoms are unrecognized by others (20). This underscores a critical clinical gap: prior management strategies have often prioritized visible lesions over subjective symptoms, yet our data suggest that neglecting the latter may perpetuate psychological distress in rosacea patients (21).

Our analysis of 32 patients receiving symptomatic treatment and 64 matched controls (carvedilol alone) further validates the clinical value of addressing subjective symptoms. After 6 weeks of treatment, the targeted group showed significantly greater improvements in global disease severity (IGA, PSA scores), subjective symptoms (burning, dryness, pruritus), anxiety (HADS-A), depression (HADS-D), and quality of life (DLQI) compared to controls. These results extend previous research, which has only linked reduced subjective symptoms to improved quality of life, by demonstrating a direct benefit for psychological status. The efficacy of the intervention may be attributed to the multimodal action of the added treatments. By resolving the sensory distress that drives anxiety, targeted treatment breaks the “symptom-anxiety cycle,” a finding that supports the integration of subjective symptom management into standard rosacea care. Importantly, the superior improvement in IGA and PSA scores in the targeted group suggests that addressing subjective symptoms also enhances perceived and objective overall disease control. This implies that subjective symptoms are not merely secondary to visible lesions but play an active role in shaping patients’ experience of disease severity. Clinically, this means that treating only visible symptoms may leave patients with unmet needs, whereas integrating subjective symptom relief can lead to more holistic recovery.

A global study has shown that subjective facial symptoms and sensory experiences are significantly associated with the burden of skin diseases (22). The study found that individuals with more severe subjective symptoms spend more time on daily skincare routines. This indicates that the impact of subjective symptoms on patients’ lives should not be underestimated, and such symptoms may also exert a certain influence on the diagnosis and treatment of rosacea patients. This study offers three key clinical takeaways. First, subjective symptoms should be systematically assessed in rosacea patients, alongside visible phenotypes. Clinicians should proactively inquire about burning, dryness, and pruritus, and use tools like the HADS to screen for anxiety—particularly in ETR patients, who exhibited higher baseline anxiety in our cohort (*p* = 0.03). Second, treatment regimens should be individualized to address subjective symptoms: for patients with moderate-to-severe symptoms, adding symptomatic treatment like neurotrophic agents or antihistamines to conventional therapy may improve both physical and psychological outcomes (23). Third, multidisciplinary collaboration, such as dermatology and psychology may be beneficial for patients with comorbid anxiety, as targeted symptom relief alone may not fully resolve longstanding psychological distress (24).

To translate these findings into practice, we propose a practical algorithm for clinicians: First, systematic assessment of subjective symptoms (burning, dryness, pruritus) should be performed at every visit using simple, validated tools such as a 0–10 visual analog scale, alongside the standard evaluation of visible signs. Second, clinicians should proactively inquire about these sensory symptoms, particularly in ETR patients who may be at higher risk for anxiety, even when objective signs appear well-controlled. Third, for patients reporting moderate-to-severe subjective symptoms, we recommend considering adjunctive symptomatic treatments and screening for anxiety using the HADS-A, as this may identify individuals who could benefit from integrated dermatological and psychological support.

This study has several limitations that should be acknowledged. First, the retrospective cross-sectional design inherently precludes establishing causal relationships between subjective symptoms, treatment, and anxiety, and may introduce selection and information bias. Second, the treatment comparison was a non-randomized, observational sub-analysis, and despite rigorous matching, the possibility of residual confounding by unmeasured factors cannot be excluded. Therefore, the findings should be interpreted as associations. Third, the sample size of the symptom-targeted treatment group was relatively small (*n* = 32), and the heterogeneity of the additional medications prevents us from ascertaining the specific contribution of each drug to the observed improvements. Fourth, the single-center setting may limit the generalizability of our findings. Future multicenter, prospective, randomized controlled trials with larger sample sizes are warranted to validate our findings and to dissect the specific effects of individual symptomatic therapies. Finally, the substantial imbalance in subgroup sizes, particularly the small number of patients with phymatous rosacea (*n* = 10), precludes robust statistical comparisons and the findings related to this subtype should be interpreted as purely descriptive. Future studies with larger and more balanced cohorts are needed to validate subtype-specific differences.

Future research should address these gaps by conducting multicenter, prospective randomized controlled trials (RCTs) with larger sample sizes and longer follow-up to validate the efficacy of targeted symptomatic treatment. Additionally, mechanistic studies exploring the neurobiological links between subjective symptoms and anxiety could identify novel therapeutic targets. Finally, investigating the impact of combined dermatological and psychological interventions may further improve outcomes for rosacea patients with comorbid anxiety.

## 5 Conclusion

Subjective symptoms are independent risk factors for anxiety in rosacea patients, and targeted treatment for these symptoms is associated with significant improvements in overall disease status, anxiety levels, and quality of life. By prioritizing subjective symptom assessment and management, clinicians can better address the physical and psychological burdens of rosacea, ultimately improving patient well-being.

## Data Availability

The raw data supporting the conclusions of this article will be made available by the authors, without undue reservation.

## References

[1] Alexis AF Callender VD Baldwin HE Desai SR Rendon MI Taylor SC. Global epidemiology and clinical Spectrum of Rosacea, highlighting skin of color: review and clinical practice experience. J Am Acad Dermatol. (2019) 80:1722–9. doi: 10.1016/j.jaad.2018.08.049, 30240779

[2] Barakji YA Rønnstad ATM Christensen MO Zachariae C Wienholtz NKF Halling A-S . Assessment of frequency of Rosacea subtypes in patients with Rosacea: a systematic review and Meta-analysis. JAMA Dermatol. (2022) 158:617–25. doi: 10.1001/jamadermatol.2022.0526, 35385049 PMC8988027

[3] Baldwin HE Harper J Baradaran S Patel V. Erythema of Rosacea affects health-related quality of life: results of a survey conducted in collaboration with the National Rosacea Society. Dermatol Ther. (2019) 9:725–34. doi: 10.1007/s13555-019-00322-5, 31512178 PMC6828914

[4] Dai R Lin B Zhang X Lou Y Xu S. Depression and anxiety in Rosacea patients: a systematic review and Meta-analysis. Dermatol Ther. (2021) 11:2089–105. doi: 10.1007/s13555-021-00613-w, 34657997 PMC8611151

[5] Incel Uysal P Akdogan N Hayran Y Oktem A Yalcin B. Rosacea associated with increased risk of generalized anxiety disorder: a case-control study of prevalence and risk of anxiety in patients with Rosacea. An Bras Dermatol. (2019) 94:704–9. doi: 10.1016/j.abd.2019.03.002, 31789266 PMC6939083

[6] Chang H-C Huang Y-C Lien Y-J Chang Y-S. Association of Rosacea with depression and anxiety: a systematic review and Meta-analysis. J Affect Disord. (2022) 299:239–45. doi: 10.1016/j.jad.2021.12.008, 34879261

[7] Mar K Rivers JK. The mind body connection in dermatologic conditions: a literature review. J Cutan Med Surg. (2023) 27:628–40. doi: 10.1177/12034754231204295, 37898903 PMC10714694

[8] Chen M Deng Z Huang Y Li J. Prevalence and risk factors of anxiety and depression in Rosacea patients: a cross-sectional study in China. Front Psych. (2021) 12:659171. doi: 10.3389/fpsyt.2021.659171, 34220573 PMC8244786

[9] Li J Wei J Zhang M Kong M Xie L Wan M . Transcutaneous auricular Vagus nerve stimulation treatment for Erythematotelangiectatic Rosacea: a randomized clinical trial. JAMA Dermatol. (2025) 161:e253796. doi: 10.1001/jamadermatol.2025.3796, 41060641 PMC12509084

[10] Gether L Overgaard LK Egeberg A Thyssen JP. Incidence and prevalence of Rosacea: a systematic review and Meta-analysis. Br J Dermatol. (2018) 179:282–9. doi: 10.1111/bjd.16481, 29478264

[11] Schaller M Dirschka T Lonne-Rahm S-B Micali G Stein-Gold L Tan J . The importance of assessing burning and stinging when managing rosacea: a review. Acta Derm Venereol. (2021) 101:356. doi: 10.2340/actadv.v101.356 34643244 PMC9425614

[12] Scharschmidt TC Yost JM Truong SV Steinhoff M Wang KC Berger TG. Neurogenic Rosacea: a distinct clinical subtype requiring a modified approach to treatment. Arch Dermatol. (2011) 147:123–6. doi: 10.1001/archdermatol.2010.413, 21242409 PMC3692271

[13] Seetan K Gablan M Alnaimi M Alhazaimeh D Younes MB Alnaimi A . Assessment of depressive and anxiety symptoms and health-related quality of life in Rosacea patients: a case-control study. Dermatol Res Pract. (2024) 2024:5532532. doi: 10.1155/drp/5532532, 39720781 PMC11668539

[14] Aedo G Chahuán M Gatica E Herrera I Parada LF Seguel A . Managing a burning face: clinical manifestations and therapeutic approaches for neurogenic Rosacea. Int J Mol Sci. (2025) 26:2366. doi: 10.3390/ijms26052366, 40076987 PMC11901027

[15] Gallo RL Granstein RD Kang S Mannis M Steinhoff M Tan J . Standard classification and pathophysiology of Rosacea: the 2017 update by the National Rosacea Society expert committee. J Am Acad Dermatol. (2018) 78:148–55. doi: 10.1016/j.jaad.2017.08.037, 29089180

[16] Schaller M Almeida LMC Bewley A Cribier B Del Rosso J Dlova NC . Recommendations for Rosacea diagnosis, classification and management: update from the global ROSacea COnsensus 2019 panel. Br J Dermatol. (2020) 182:1269–76. doi: 10.1111/bjd.18420, 31392722 PMC7317217

[17] Li J Tang J-Y Fu J Zhang M-W Wan M Chen D-W . Carvedilol ameliorates persistent erythema of Erythematotelangiectatic Rosacea by regulating the status of anxiety/depression. J Dermatol. (2022) 49:1139–47. doi: 10.1111/1346-8138.16525, 35904063

[18] Zigmond AS Snaith RP. The hospital anxiety and depression scale. Acta Psychiatr Scand. (1983) 67:361–70. doi: 10.1111/j.1600-0447.1983.tb09716.x, 6880820

[19] Finlay AY Khan GK. Dermatology life quality index (DLQI)--a simple practical measure for routine clinical use. Clin Exp Dermatol. (1994) 19:210–6. doi: 10.1111/j.1365-2230.1994.tb01167.x, 8033378

[20] Oussedik E Bourcier M Tan J. Psychosocial burden and other impacts of Rosacea on patients’ quality of life. Dermatol Clin. (2018) 36:103–13. doi: 10.1016/j.det.2017.11.005, 29499793

[21] Heisig M Reich A. Psychosocial aspects of Rosacea with a focus on anxiety and depression. Clin Cosmet Investig Dermatol. (2018) 11:103–7. doi: 10.2147/CCID.S126850, 29551906 PMC5844253

[22] Zeidler C Kupfer J Dalgard FJ Bewley A Evers AWM Gieler U . Dermatological patients with itch report more stress, stigmatization experience, anxiety and depression compared to patients without itch: results from a European multi-Centre study. J Eur Acad Dermatol Venereol. (2024) 38:1649–61. doi: 10.1111/jdv.19913, 38468596

[23] Tan J Steinhoff M Bewley A Gieler U Rives V. Characterizing high-burden Rosacea subjects: a multivariate risk factor analysis from a global survey. J Dermatol Treat. (2020) 31:168–74. doi: 10.1080/09546634.2019.1623368, 31120382

[24] Moustafa F Lewallen RS Feldman SR. The psychological impact of Rosacea and the influence of current management options. J Am Acad Dermatol. (2014) 71:973–80. doi: 10.1016/j.jaad.2014.05.036, 24993600

