# Supplementary material for: Subjective symptoms are associated with anxiety in patients with rosacea: a cross-sectional study
Source: Front Public Health. 2026 Aug 13;14:1886167. doi: 10.3389/fpubh.2026.1886167 (PMC13518192; doi:10.3389/fpubh.2026.1886167)
Supplement: Supplementary file 1 [file Table_1.DOCX]

| **Characteristic** | **Without Anxiety（123）** | **With Anxiety（359）** | **p value** |
| --- | --- | --- | --- |
| **Sex** |  |  |  |
| male | 51 | 130 | 0.3 |
| female | 72 | 229 |  |
| **Age** | 40.62（10.33） | 40.61（11.46） | 0.83 |
| **BMI** | 22.47(4.28) | 23.02(5.14) | 0.12 |
| **Flushing** |  |  |  |
| None or mild | 21(17.1%) | 58(16.2%) | 0.813 |
| Moderate to severe | 102(82.9%) | 301(83.8%) |  |
| **Telangiectasia** |  |  |  |
| None or mild | 104(84.6%) | 257(72.0%) | **0.005** |
| Moderate to severe | 19 (15.4%) | 100(28.0%) |  |
| **Persistent Erythema** |  |  |  |
| None or mild | 24(19.7%) | 47(13.1%) | **0.077** |
| Moderate to severe | 98(80.3%) | 312(86.9%) |  |
| **Papulopustule** |  |  |  |
| None or mild | 97(78.9%) | 281(78.5%) | 0.931 |
| Moderate to severe | 26(21.1%) | 77(21.5%) |  |
| **Rhinophyma** |  |  |  |
| None or mild | 119(96.7%) | 345(96.1%) | 0.744 |
| Moderate to severe | 4(3.3%) | 14(3.9%) |  |
| **Burning** |  |  |  |
| None or mild | 88(71.5%) | 208(57.9%) | **0.007** |
| Moderate to severe | 35 (28.5%) | 151 (42.1%) |  |
| **Stinging** |  |  |  |
| None or mild | 118(95.9%) | 312(86.9%) | **0.005** |
| Moderate to severe | 5(4.1%) | 47(13.1%) |  |
| **Edema** |  |  |  |
| None or mild | 118(95.9%) | 315(87.7%) | **0.009** |
| Moderate to severe | 5(4.1%) | 44(12.3%) |  |
| **Dryness** |  |  |  |
| None or mild | 113(91.9%) | 297(77.9%) | **0.001** |
| Moderate to severe | 10(8.1%) | 79(22.1%) |  |
| **Pruritus** |  |  |  |
| None or mild | 116(94.3%) | 305(85.0%) | **0.007** |
| Moderate to severe | 7(5.7%) | 54(15.0%) |  |
| **DLQI** |  |  |  |
| None | 16(13.0%) | 4(1.1%) |  |
| Mild | 35(28.5%) | 25(6.9%) | **<0.001** |
| Moderate | 22(17.9%) | 82(22.8%) |  |
| Severe | 35(28.5%) | 158(44.0%) |  |
| Extremely Severe | 15(12.2%) | 90(25.1%) |  |

**Supplementary Table 1**

The univariate analysis of rosacea symptoms in two patient groups with or without comorbid anxiety disorders.

Note: Bold indicates p-values with statistical significance (p<0.05)
